# Integrative RNA- and miRNA-Profile Analysis Reveals a Likely Role of BR and Auxin Signaling in Branch Angle Regulation of *B. napus*

**DOI:** 10.3390/ijms18050887

**Published:** 2017-05-08

**Authors:** Hongtao Cheng, Mengyu Hao, Wenxiang Wang, Desheng Mei, Rachel Wells, Jia Liu, Hui Wang, Shifei Sang, Min Tang, Rijin Zhou, Wen Chu, Li Fu, Qiong Hu

**Affiliations:** 1 Oil Crops Research Institute of Chinese Academy of Agricultural Sciences/Key Laboratory for Biological Sciences and Genetic Improvement of Oil Crops, Ministry of Agriculture, Wuhan 430062, China; cht1306@163.com (H.C.); moluozhige@163.com (M.H.); wangwx8@163.com (W.W.); deshengmei@caas.cn (D.M.); liujia02@caas.cn (J.L.); 18062011466@163.com (H.W.); 15652142445@163.com (S.S.); tangmin924@163.com (M.T.); weiming3130@163.com (R.Z.); chuwen100@163.com (W.C.); irene87319@126.com (L.F.); 2 John Innes Centre, Norwich Research Park, Norwich NR4 7UH, UK; rachel.wells@jic.ac.uk

**Keywords:** miRNA, branch angle, *Brassica napus*, deep sequencing, auxin, brassinosteroid

## Abstract

Oilseed rape (*Brassica napus* L.) is the second largest oilseed crop worldwide and one of the most important oil crops in China. As a component of plant architecture, branch angle plays an important role in yield performance, especially under high-density planting conditions. However, the mechanisms underlying the regulation of branch angle are still largely not understood. Two oilseed rape lines with significantly different branch angles were used to conduct RNA- and miRNA-profiling at two developmental stages, identifying differential expression of a large number of genes involved in auxin- and brassinosteroid (BR)-related pathways. Many auxin response genes, including *AUX1*, *IAA*, *GH3*, and *ARF*, were enriched in the compact line. However, a number of genes involved in BR signaling transduction and biosynthesis were down-regulated. Differentially expressed miRNAs included those involved in auxin signaling transduction. Expression patterns of most target genes were fine-tuned by related miRNAs, such as miR156, miR172, and miR319. Some miRNAs were found to be differentially expressed at both developmental stages, including three miR827 members. Our results provide insight that auxin- and BR-signaling may play a pivotal role in branch angle regulation.

## 1. Introduction

Rapeseed is one of the most important oil crops for a large part of the world. In the context of decreasing arable lands and increasing population, maximizing the yield potential of crops is a principal goal for plant breeders. One strategy to increase yield is to implement high-density planting on limited land [1], enhancing the requirement for efficient resource capture and utilization [2,3,4]. The space occupied by plants above ground is mainly determined by the number, length, and angle of the lateral branches [5,6,7]. Amongst all plant architecture traits, leaf angle or branch angle has been demonstrated to be essential for high-density planting [2,3]. Tiller or branch angle has been well investigated among crops including rice and maize, owing to its’ agronomic importance [6,7]. Moreover, the growth angle of branches and other lateral organs are also emerging as important topics for plant developmental research [8].

Remarkable progress has been made regarding the identification of quantitative trait loci (QTL) and genes for tiller or leaf angle over the last few decades. Those identified in rice include *LAZY1*, *TILLER ANGLE CONTROL1*, *PROSTRATE GROWTH1*, and *LOOSE PLANT ARCHITECTURE1* [9,10,11,12]. Moreover, orthologous genes in other species, e.g., *ZmTAC1*, *ZmLAZY1* in maize, and *AtLAZY1* and *AtLPA1* in Arabidopsis, have also been shown to be involved in leaf or branch angle regulation [13,14].

Asymmetric distribution of auxin has long been regarded as the main factor that affects shoot gravitropism [15,16], a key component in controlling branch orientation. Changes in the expression levels of genes involved in auxin synthesis or signaling transduction have been shown to result in differences in the tiller angle and gravitropic response [8]. In addition to auxin, brassinosteroids (BRs) have been reported to be involved in leaf angle determination [17].

MicroRNAs (miRNAs) are small non-coding 20–24 nt RNAs that can repress target gene expression [18]. Multiple studies have shown the importance of miRNA regulation of corresponding target genes in a wide range of plant developmental processes. In Arabidopsis, the vegetative to reproductive phase transition is regulated by miR156 and miR172 [19]. miR319, which is known to target TCP (TEOSINTE-BRANCHED/CYCLOIDEA/PCF) transcription factors, has been shown to regulate leaf development and morphogenesis by controlling the plant organ fate [20].

In recent years, many researchers have illustrated the interaction between miRNAs and phytohormone responses, widely improving the understanding of miRNA-hormone interactions in plant development [21]. miR393 and the corresponding target regulatory module have been shown to influence many aspects of plant developmental processes via auxin, such as root architecture and leaf development [22,23,24,25,26]. miR164 can degrade the mRNA of transcription factor *NAC1*, down-regulating auxin response signals and inhibiting the growth of lateral roots [27]. Auxin response genes *ARF6* and *ARF8* are predicted to be targets of miR167 in a wide range of species [28,29,30]. Overexpression of rice Os-miR160 increased the tiller angle while decreasing effective tiller numbers [31]. Different from other miRNAs, miR390 directed *TAS3* cleavage to trigger trans-acting small interfering RNAs (ta-siRNA) biogenesis and subsequently inhibit *ARF2/ARF3/ARF4* [32,33].

Although much is known about the control of leaf and tiller angle in Arabidopsis and rice, only limited studies on branch angle determination in *B. napus* have been reported. Genomic regions harboring QTLs for branch angle were detected by association mapping with oilseed rape germplasm lines. Orthologous genes involved in auxin signaling and gravitropism response were perceived within the QTL regions [34,35]. QTL-seq bulk segregant analysis (BSA) of an F_2_ population identified *BnaYUCCA6*, a gene implicated in auxin synthesis, as a candidate for branch angle regulation [36].

To gain more insights into the mechanisms of branch angle regulation in *B. napus*, we profiled genome-wide gene and miRNA expression in two lines exhibiting significantly different branch angles by high-throughput sequencing technology. Numerous genes and some miRNAs showing differential expression were detected. Differentially expressed gene (DEG) analysis showed that a large number of DEGs were involved in auxin- and BR- related pathways. Some targets of differential expressed miRNAs were also found to be involved in auxin signaling and developmental pathways. Our results provide further insight into the putative role of auxin- and BR-related genes in branch angle regulation.

## 2. Results

### 2.1. Differentially Expressed Gene Analysis by RNA-Seq

**Figure 1:**
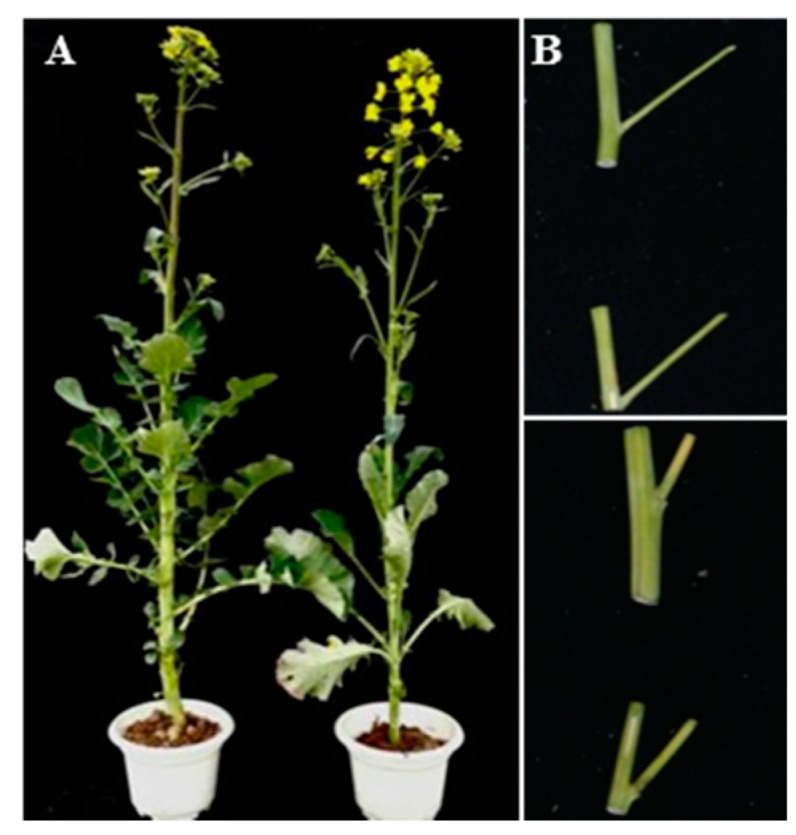
Phenotypes of 6098B and Purler showing variation in branch angle. (**A**) 6098B and Purler at early flowering. (**B**) **Upper panel**: 6098B showing lax branch angle (52°), **lower panel**: erect branch angle of Purler (22°).

**Figure 2:**
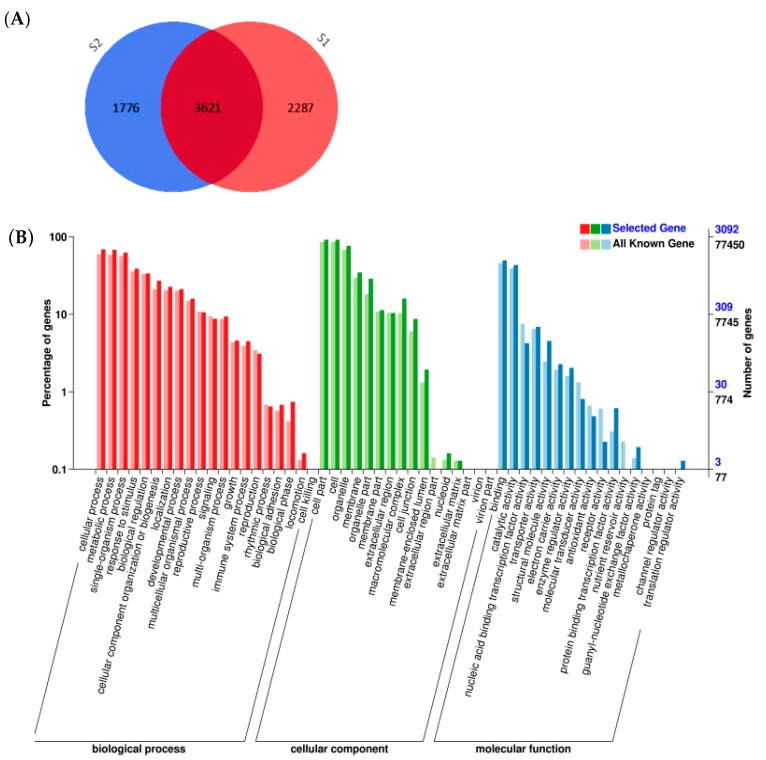
Number and functional classification of differentially expressed genes (DEGs). (**A**) The number of DEGs detected between 6098B and Purler at bolting (blue), early flowering (light pink), and both (dark pink) developmental stages. (**B**) Functional classification of DEGs identified at both development stages by Gene Ontology (GO) categorization. Bold colours indicate the representation in the whole genome, the light colours indicate representation in the DEGs.

Two *B. napus* lines (6098B and Purler) with significantly different branch angles were selected to perform transcriptome expression profile analysis. The average branch angle of 6098B and Purler was 52 and 22 degrees, respectively (Figure 1). Samples from the branch emergence site collected at both bolting and early flowering were subjected to transcriptome sequencing (Illumina HiSeq2000, Illumina, San Diago, CA, USA) generating ~46.86 million and ~45.36 million raw reads from 6098B and Purler, respectively. After quality filtration, 23.06 Gb (with Q30 ≥ 91.45%) of data remained. The mapping ratio of all reads to the reference genome was about 82.7% [37]. At bolting, 5908 DEGs were detected between 6098B and Purler with a fold change ≥2 (FDR < 0.01). We also found 5397 DEGs between 6098B and Purler at the early flowering stage (Tables S1 and S2). Of these, 3621 genes were found to be differentially expressed at both developmental stages (Figure 2A, Table S3).

### 2.2. Functional Classification by Gene Ontology and Metabolic Pathway Analysis

Gene Ontology (GO) annotation analysis of the 3621 DEGs identified at both developmental stages showed that DEGs were divided into 51 different groups, which could be further divided into three main classifications: cellular component (29%), molecular function (27%), and biological process (44%) (Figure 2B). Significant differences between the distributions of GO terms for the DEGs compared to the whole genome were used to reveal the functional significance of the changes observed. Within the biological process classification, biological adhesion, biological phase, and locomotion were detected to be overrepresented in the DEGs compared to the whole genome (Figure 2B). Within cellular components, the GO terms including macromolecular complex, cell junction, and nucleoid were identified to be enriched (Figure 2B). For molecular function, the analysis identified the enrichment of nuclear and protein binding transcription factor activity and receptor activity.

**Figure 3:**
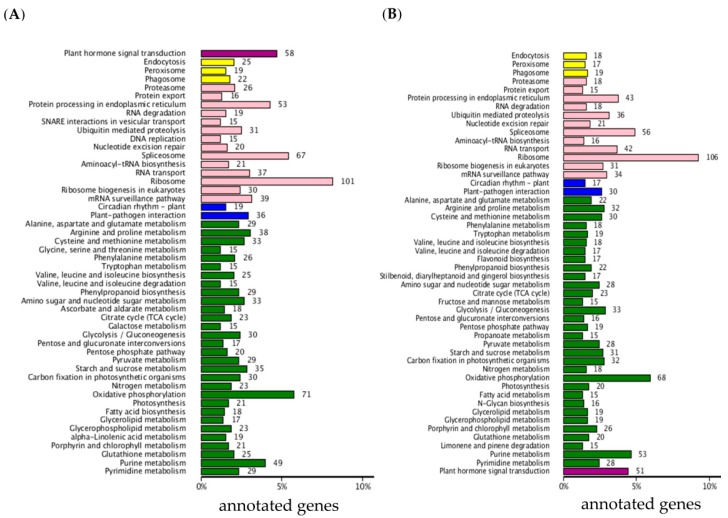
Pathway analysis of DEGs based on the KEGG database. (**A**) Analysis of DEGs at the bolting and (**B**) early flowering stages. The *X*-axis indicates the pathways, the *Y*-axis indicates the numbers of annotated genes.

**Figure 4:**
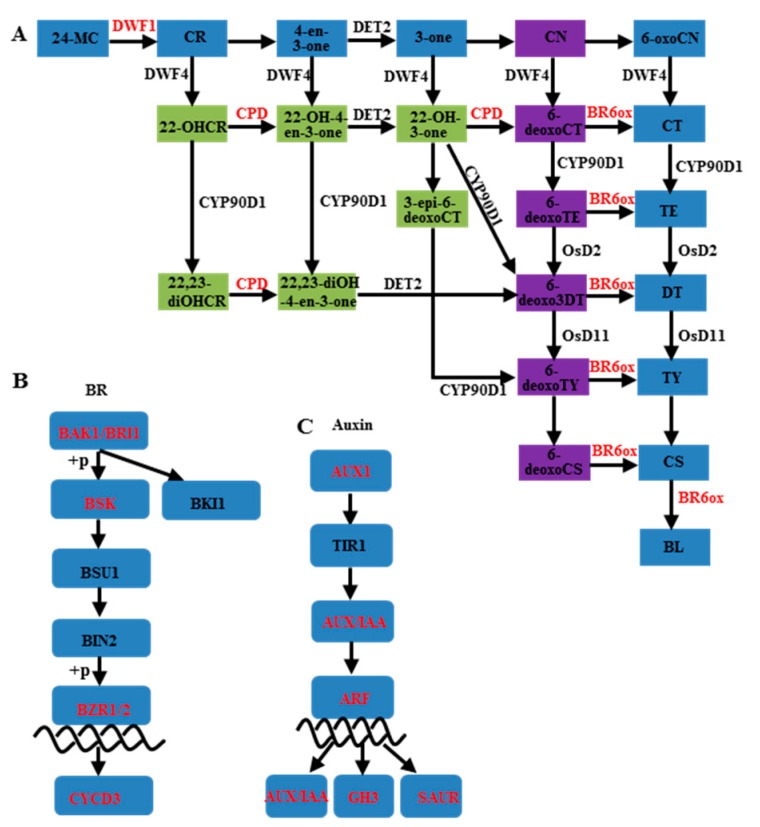
Schematic diagrams of (**A**) the BR biosynthesis pathway, (**B**) BR signal transduction pathway, and (**C**) auxin signaling transduction pathway. Genes marked in red indicate those differentially expressed between the two lines.

**Figure 5:**
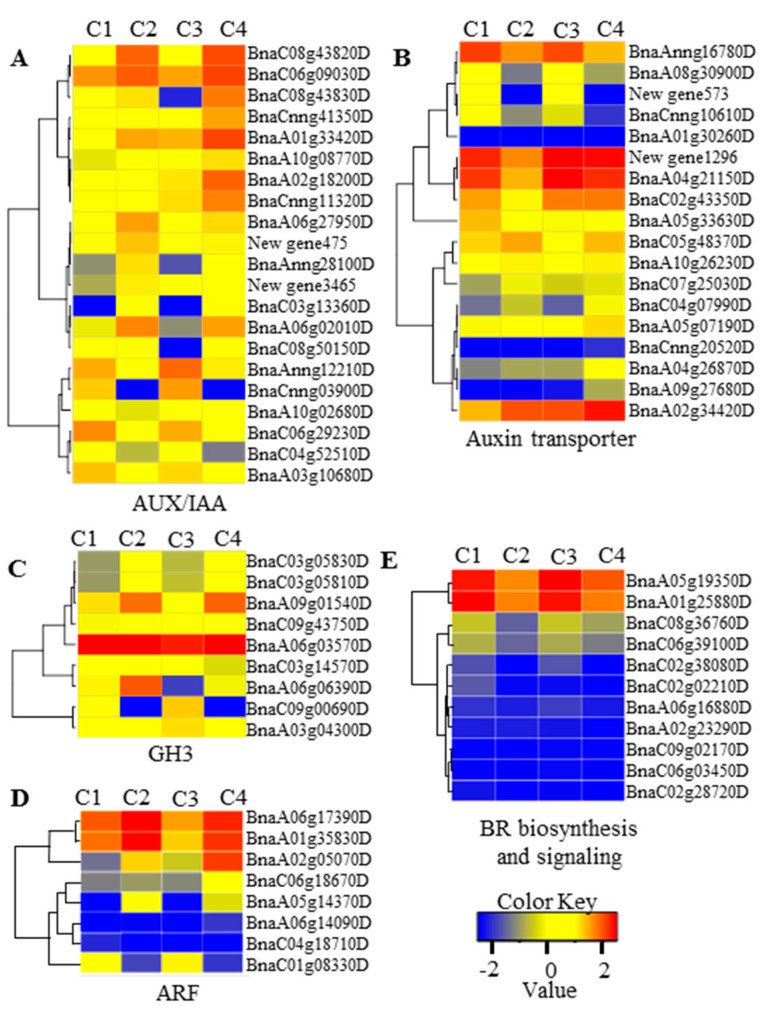
Heat maps of DEGs related to the auxin and BR signaling pathways showing (**A**–**D**) the cluster of DEGs involved in auxin signaling transduction and (**E**) the cluster of DEGs involved in BR biosynthesis and transduction. Tissue samples from 6098B (C1, C3) and Purler (C2, C4) at the bolting and early flowering stages, respectively. Color key represents log2 transformed FPKM (fragments per kilobase of exon per million fragments mapped) values, from low (blue) to high (red) expression.

Using all DEGs between 6098B and Purler at the two development stages, 3279 genes were mapped to the KEGG (Kyoto Encyclopedia of Genes and Genomes) database. The metabolic pathways of these DEGs were classified into 50 different terms (Figure 3), with ribosome and oxidative phosphorylation pathways accounting for a large proportion of the DEGs. It should be noted that about 58 and 51 genes were grouped into plant hormone signaling pathways at the bolting and early flowering stages, respectively. Detailed analysis showed that DEGs grouped into the plant hormone signaling pathway category were mainly classified as being involved in auxin and BR signaling transduction. Some DEGs were found to be enriched in the BR biosynthesis pathway. Three homologs of *CYP85A* and *CYP90A*, which encode key enzymes in BR synthesis, were differentially expressed in 6098B and Purler at both development stages (Figure 4A and Figure 5E). BR6OX (CYP85A) regulates multiple C-6 oxidation steps during BR biosynthesis (Figure 4A). Two genes encoding the putative BR biosynthetic enzyme, DWRF1, were identified to be down-regulated in Purler compared to 6098B (Figure 5E). DWRF1 catalyzes the conversion of 24-methylenecholesterol (24-MC) to campesterol (CR), which determines the first step of BR biosynthesis (Figure 4A). Other genes involved in the BR signaling pathway, including *BRI1*, *BSK*, *BZR1*, and *CYCD3*, were also found to be down-regulated in Purler (Figure 4B and Figure 5E). It is noted that DEGs including auxin signaling pathway genes, *AUX1*, *IAA*, *GH3*, and *ARF*, were found to be enriched (Figure 4C and Figure 5A–D). Most of the *IAAs*, *GH3s*, and *ARFs* genes were up-regulated in Purler at both bolting and early flowering (Figure 5A–D). These genes have been demonstrated to be the important components of the auxin signaling pathway (Figure 4C). Some polar auxin transport genes including *ABCs* and *BIGs* were also differential expressed (Figure 4B). Due to the close relationship between the lateral organ angle and phytohormones, genetic manipulation of these genes is necessary for further elucidating the mechanism underlying branch angle regulation.

### 2.3. Differentially Expressed miRNA in 6098B and Purler

**Figure 6:**
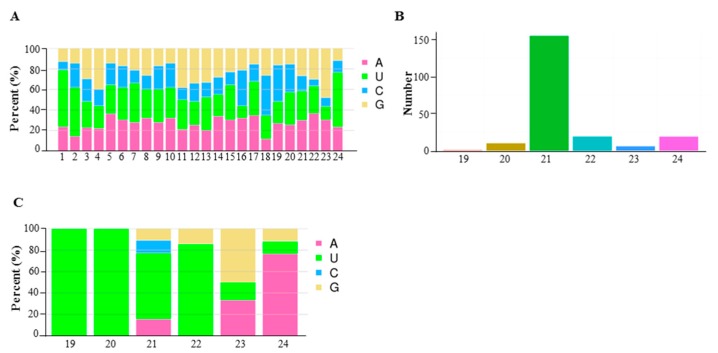
Nucleotide preference of small RNAs in *B. napus* showing (**A**) nucleotide preference at each position; (**B**) number of 19- to 24-nucleotide (nt) small RNAs in all the identified small RNAs and (**C**) the first nucleotide bias of 19- to 24-nucleotide (nt) small RNA.

miRNAs are well recognized as important regulators of plant development via transcription cleavage or translation repression. Plant hormone, typically auxin, signaling has been shown to be influenced by miRNAs, therefore we also conducted siRNA sequencing of 6098B and Purler at both developmental stages. After removing adaptors and low quality reads, ~7.6 and 9 M (6098B) and ~7.1 and 7.3 M (Purler) clean reads, with lengths from 18 to 30 bp, were obtained from the bolting and early flowering stages, respectively. A total of 13.18 M annotated reads were mapped to the *B. napus* genome, ~3.7, 4.4, 2.4, and 2.7 M reads in four samples. The analysis identified 202 miRNAs, including 85 known and 111 new miRNAs (Tables S4 and S5). Nucleotide composition analysis showed that cytosine (C) was the least represented base within the miRNAs (Figure 6A). Nucleotide composition and base preference of the newly detected miRNAs were consistent with that of known miRNAs (Figure 6A). miRNAs of 21-nucleotide length were the most abundant of the newly identified miRNAs, similar to the length distribution of known miRNAs (Figure 6B). Newly identified miRNAs showed a preference for adenosine (A) at the 1, 2, and 24 position, however, those miRNAs with mature lengths of 19–22 bp started with uridine (U) (Figure 6C).

**Figure 7:**
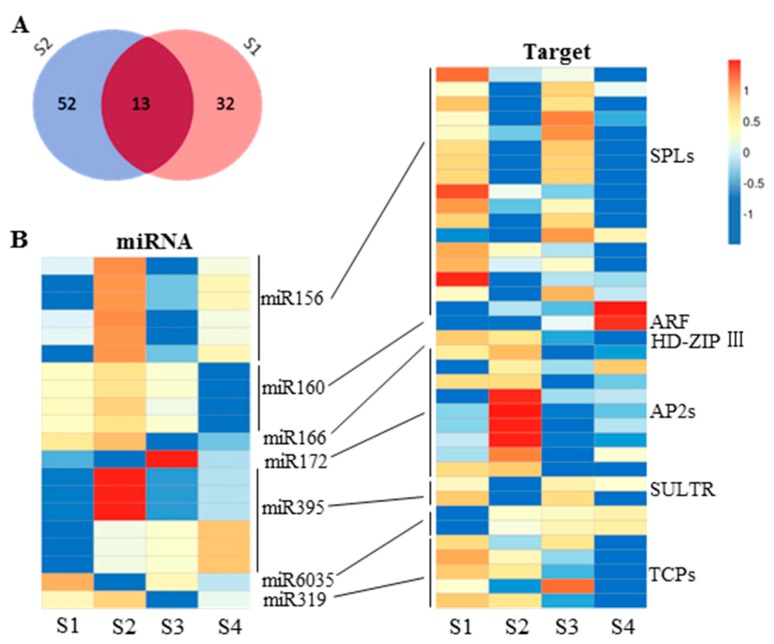
Number of differentially expressed miRNAs and the expression profile of miRNA-targets. (**A**) The number of differentially expressed miRNAs detected between 6098B and Purler at bolting (blue), early flowering (light pink), and both (dark pink) developmental stages. (**B**) The heat map of differentially expressed miRNAs and targets showing greater than 2-fold change between 6098B (S1, S3) and Purler (S2, S4) at bolting or early flowering. SPL (SQUAMOSA promoter binding protein-like), ARF (auxin response factor), HD-ZIP III (Homeodomain leucine zipper III), AP2 (APETALA2), SULTR (sulfate transporter), TCP (TEOSINTE-BRANCHED/CYCLOIDEA/PCF). Color key represents log2 transformed FPKM values, from low (blue) to high (red) expression.

Examination of miRNA expression dynamics in 6098B and Purler identified 45 and 65 differentially expressed miRNAs at the two developmental stages, respectively (Figure 7A, Tables S6 and S7). Besides miR1140 and miR827, all known miRNAs differentially expressed at bolting had higher expression in Purler. This included three members of the miR156 family and six members of the miR395 family. By contrast, most differentially expressed known miRNAs were down-regulated at early flowering. Besides three members from the miRNA156 family and miRNA166f, other known miRNAs accumulated less in Purler than 6098B at this stage. Comparison of miRNAs differentially expressed at the two developmental stages identified 13 novel miRNAs with consistent differential expression both at the bolting and early flowering stages (Table S8). Mature sequences analysis revealed that some miRNA members belong to one miRNA family [38]. For example, three of the thirteen miRNAs are members of the miR827 family. According to previous work, another three members belong to miRX115.1 [38]. Seven other differential expressed novel miRNAs had not been previously detected.

To gain further insight into putative functions, targets of the differentially expressed miRNAs implicated in branch angle regulation were predicted. Two predicted targets of miRX215 were the orthologues of the Arabidopsis gene encoding organellar (peroxisome, glyoxysome) 3-ketoacyl-CoA thiolase. Three targets of miRX115.1 had previously been identified and verified by degradome sequencing [38]. Orthologues of these targets in Arabidopsis encode an ATP-dependent caseinolytic (Clp) protease. According to the results from Shen et al. (2015), nine putative targets of miR827 were found in *B. napus*. Among all targets of miR827, two genes encoding VILLIN proteins were confirmed by degradome sequencing [38]. Among all these miRNA targets, only VILLIN protein has been demonstrated to play an important role in plant architecture regulation by affecting polar auxin transport [39,40].

### 2.4. Validation of Differential Gene and miRNA Expression by semi-RT-PCR and qRT-PCR

**Figure 8:**
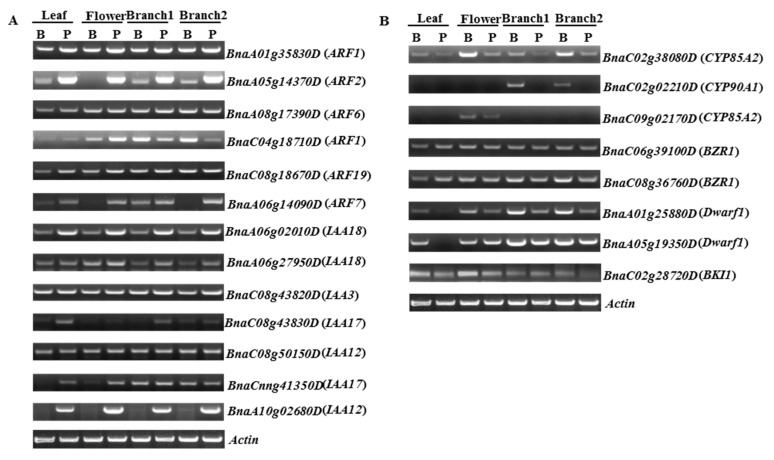
Validation of DEGs involved in (**A**) auxin signaling pathway and (**B**) BR signaling transduction and biosynthesis, by RT-PCR in 6098B (B) and Purler (P). Actin was amplified with 27 cycles, other genes were amplified with 34 cycles.

Differentially expressed genes involved in BR or auxin biosynthesis and signaling transduction were selected to verify RNA-seq observations by semi-quantitative RT-PCR. Leaf and flower bud samples from the two lines were added for comparison. The expression patterns observed were consistent with those determined by RNA-seq, thereby validating the results obtained. Four ARF gene family and seven IAA gene family homologues showed clearly higher abundance in Purler than in 6098B (Figure 8A). The transcription level of BnaC04g18710D, an orthologue of ARF1, was detected to be lower in Purler at both developmental stages. However, the expression level of BnaC04g18710D accumulated more in Purler than in 6098B in leaf and flower samples. Genes predicted to be involved in the BR biosynthesis or signaling pathway were down-regulated in Purler when compared to 6098B, the opposite of the expression pattern of the auxin related genes (Figure 8B). For example, the expression of BR biosynthesis gene orthologues, including *CYP85A*, *CYP90A*, and *DWARF*, was clearly down-regulated in Purler.

**Figure 9:**
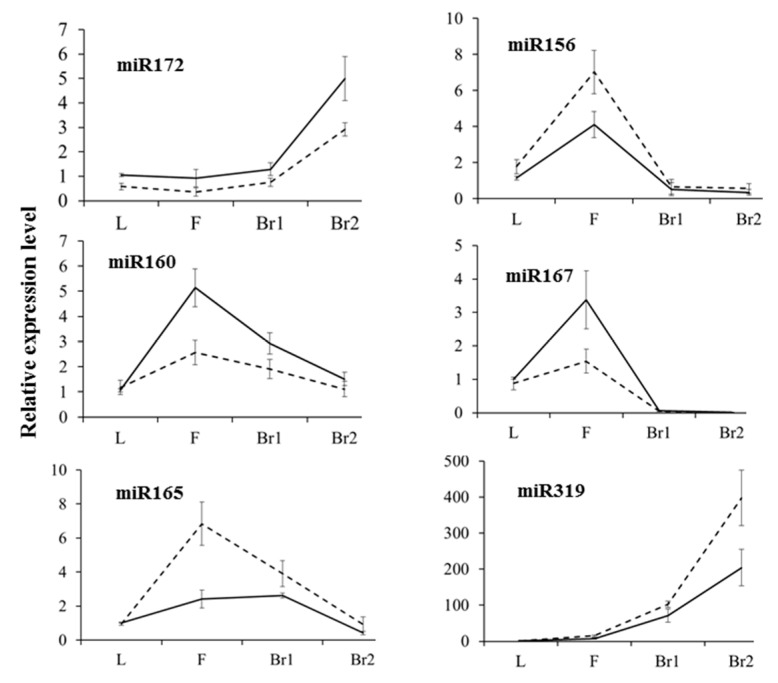
Validation of selected differentially expressed miRNAs by qRT-PCR in the leaf (L), flower bud (F), branching site at bolting (Br1), and branching site at early flowering (Br2) in 6098B (solid line) and Purler (dotted line), performed on the same tissue samples used for verifying gene expression. Data represent means (three biological replicates) ± standard deviation.

As a large number of miRNAs were identified to be differentially expressed between the two lines, the most interesting miRNAs were selected for validation by stem-loop qRT-PCR. Expression patterns of most miRNAs were correlated with the results obtained via miRNA sequencing. For example, the expression level of miR156 was lower in 6098B in all four tissue samples (Figure 7 and Figure 9). The expression levels of miR172 and miRNA160 were greater in 6098B (Figure 7 and Figure 9). Two other miRNAs, miR165 and miR319, showed increased expression in Purler at bolting (Figure 7 and Figure 9).

### 2.5. miRNA and Gene Network Analysis in 6098B and Purler

The expression levels of the majority of miRNAs were negatively associated with those of the corresponding target genes. When the expression of miRNA was higher in 6098B than that of Purler, the expression of some potential target genes was lower. For example, miR156 was up-regulated in Purler compared to 6098B, whereas the expression of most of the target SPL genes was decreased. Conjoint analysis of other represented miRNAs was also performed. The expression level of miR395 and miR319 was greater in Purler than in 6098B. The corresponding target genes of these two miRNAs, *Sulfate Transporter* (*SULT*) and *TEOSINTE-BRANCHED/CYCLOIDEA/PCF* (*TCP*), showed reduced expression. *ARF* and *AP2* were predicted to be gene targets of miR160 and miR172, respectively. These two miRNAs displayed higher expression while the targets were down-regulated (Figure 7). These results suggest that our miRNA-seq and RNA-seq data are reliable. The miRNA-gene pairs with inverse transcriptional associations may be used as potential candidates for further genetic manipulation of the plant branch angle.

## 3. Discussion

### 3.1. Branch Angle Regulation in B. napus and Other Species

Plant architecture and above ground shape are mainly determined by the number, length, and angle of branches [8]. Appropriate shoot branch angles are required to produce leaves and other organs orientated for the most efficient light interception [41]. Under high plant density, the branch angle decreases significantly [41]. Therefore, branch angle is an important component for determining the ideal plant architecture in *B. napus* as well as in other crops. Alteration of the branch angle is also considered to be useful for combined harvesting under higher plant density [41]. Though the importance of branch angle has been gradually recognized, the underlying mechanisms of branch angle regulation are still largely not understood. With the development of high throughput sequencing technology, researchers can easily obtain information on gene expression differences. Herein we ascertain the molecular mechanism of branch angle regulation in *B. napus* by RNA- and miRNA-profiling. A large number of DEGs were observed between two lines with obviously different branch angles. These included key genes involved in the biosynthesis and signaling transduction of auxin and BR. This provides substantial information for establishing the ideal plant architecture in *B. napus*.

### 3.2. Auxin and Brassinosteroids in Branch Angle and Leaf Inclination Regulation

Branch angle is considered to be relative to gravitropism (displays a gravitropic set point) and varies among species and genotypes [8]. In rice and Arabidopsis, polar auxin transport has been shown to play an important role in shoot branching. Suppressed expression of the rice polar auxin transport gene *OsPIN1* significantly increased the tiller numbers and tiller angle [42]. Rice plants overexpressing *OsPIN2* and *OsPIN3a* also displayed a larger tiller angle compared to the wild type [43,44]. Overexpression of members of the rice GH3 family, including *GH3.1*, *GH3.2*, and *GH3.8*, increased leaf inclination [45,46,47]. Both auxin and brassinosteroid signaling were reported to be involved in leaf inclination regulation [45,48,49,50]. Crosstalk between auxin and BR regulate many aspects of plant growth and development processes [51,52,53,54]. Rice auxin response factor *OsARF19* controls rice leaf angles through positively regulating *OsGH3.5* and *OsBRI1* [55]. However, the interaction between auxin and BR in regulating plant development remains elusive [56]. Osa-miR1848 has been shown to regulate phytosterol and BR biosynthesis in rice by direct mRNA cleavage of the target gene, *OsCYP51G3* [57]. Increased expression of osa-miR1848 caused dwarf plants, erect leaves, and other typical BR deficient phenotypes [57]. In our study, genes encoding enzymes for putative BR biosynthesis and signaling transduction were detected to be down-regulated in Purler compared to 6098B. In contrast, a large amount of genes involved in auxin response and signaling were identified to be up-regulated. Since Purler was more compact than 6098B, we speculate that the branch angle is modulated by enhancing the auxin pathway while suppressing the brassinosteroid pathway. Further study should establish the detailed function of genes in the key steps of the BR biosynthesis or auxin signaling pathways by genetic transformation.

### 3.3. Involvement of miRNA in Plant Architecture Establishment

miRNAs play a critical role in plant development. Transgenic plants overexpressing miR393 showed altered auxin signaling and enlarged flag leaf inclination in rice [58]. Suppressed expression of target genes *OsAFB2* and *OsTIR1* also increased inclination of flag leaf at the bolting stage [58]. Higher expression of *OsSPL14*, which is the target of miR156 in rice, promoted panicle branching and enhanced grain yield [59,60]. The maize *ZmLG1* and rice *OsLG1* gene, which is the closest homolog of *AtSPL8*, were demonstrated to control the branch angle of tassel and panicle, respectively [61,62]. Overexpression of rice Os-miR160 increased the tiller angles but decreased the number of effective tillers [31].

In our study, 45 and 65 differential expressed miRNAs were detected between Purler and 6098B at the two developmental stages, respectively. miR156, miR395, and miR166 family members were found to be more abundant in Purler than 6098B at bolting. Conversely, the *SPL* target genes of miR156 were downregulated. At the early flowering stage, we found a large number of miRNAs, including miR172, miR319, and miR160 family members, that showed reduced expression in Purler compared to 6098B. As observed with miR156/SPL, the expression level of many ARFs, predicted to be the target of miR160, were upregulated in Purler compared to 6098B. These results suggest that miR156/SPL and miR160/ARF modules may play important roles in branch angle regulation. Thirteen miRNAs have been discovered to be differential expressed at two developmental stages simultaneously, including three miR827 family members. Two Arabidopsis *VILLIN* gene homologues have been predicted to be putative targets of miR827. The quantity of AtVIN2 was identified to be significantly different between the gravity persistent signal (GPS) treatment and control, which indicated involvement in the gravitropic response [63]. Meanwhile, VLN2 has been demonstrated to regulate plant architecture both in rice and Arabidopsis [39,40]. Further study should illuminate the detailed role of miR827 in branch angle regulation as well as in the gravitropism response.

## 4. Materials and Methods

### 4.1. Plant Materials and RNA Preparation

Plant samples used for the miRNA and RNA-seq analyses were grown in the field at the Oil Crops Research Institute, Chinese Academy of Agricultural Sciences (OCRI-CAAS, Wuhan, China). Oilseed rape lines 6098B and Purler, with large and small branch angles respectively, were used for expression analysis. Tissue samples at the branch emergence site were collected at the bolting and early flowering stages. A minimum of five samples were collected for each plant for at least six individuals per line. Samples were collected at approximately the same time. All samples from each line at one development stage were mixed as a pool. Total RNA was extracted with Trizol Reagent (Invitrogen, Carlsbad, CA, USA) according to the published protocols. RNA samples were checked by Nanodrop 2000 (Thermo Fisher Scientific, Waltham, MA, USA) to test A260/A280 for protein contamination and A230/A280 for reagent contamination. We also examined the RNA integrity number using an Agilent Technologies 2100 Bioanalyzer (Agilent Technologies, Palo Alto, CA, USA).

### 4.2. Transcriptome Sequencing and Gene Expression Analysis

After checking the RNA quality, cDNA libraries were constructed and used for Illumina sequencing following previous methods [64]. Raw data was filtered by removing adapters and low quality data and the resulting clean data was aligned to the *Brassica napus* reference genome (available at: http://www.genoscope.cns.fr/brassicanapus/). Potential duplicate molecules were removed from the aligned BAM/SAM format records. FPKM (fragments per kilobase of exon per million fragments mapped) values were used to analyze gene expression by the software Cufflinks [65]. New genes were identified based on new discovered transcripts by Cufflinks and genes encoding peptides with less than 50 amino acid residues were filtered [66].

DEseq was employed to evaluate differential gene expression between 6098B and Purler [67]. Gene abundance differences between the two lines were then calculated by the ratio of FPKM values. The false discovery rate (FDR) was used to identify the threshold of the *p*-value in order to test the significance of differences. The DEGs between different samples was set as the absolute value of log2 Ratio ≥2 and FDR ≤0.001. Functional annotation was conducted by comparing genes against three protein databases by BLASTX, including the Swiss-prot database, NCBI protein database, and KEGG database. GO annotation was carried out by Blast2GO [68]. All annotated genes were mapped to the database and the numbers of genes in each GO term were determined. The annotation was then refined and enriched by using TopGo (*R* package). The enrichment of DEGs in KEGG pathways was analyzed by KOBAS software 2.0 [69]. The heat maps were drawn according to the log2 transformed FRKM values. The color key represents the log2 transformed FPKM values, from low (blue) to high (red) expression. Positive and negative expression means high and low expression level. The expression values for a given gene are normalized for each row.

### 4.3. Identification of Known and Novel miRNAs

SiRNA sequencing libraries were constructed from the RNA used for RNA-seq. Adapter and low quality reads were removed to generate clean data. Reads were trimmed and cleaned by removing sequences smaller than 18 nt or longer than 30 nt. The clean reads were aligned to several siRNA databases, including the Silva database, GtRNAdb database, Rfam database, and Repbase database. The rRNA, tRNA, snRNA (small nuclear RNA), snoRNA (small nucleolar RNA), and other ncRNA and repeats were filtered out. The remaining reads were used to detect known and novel miRNA by the miRDeep2 software [70]. New miRNAs were predicted by comparing with known miRNAs from miRBase (available at: http://www.mirbase.org/). A new miRNA secondary structure was predicted by RNAfold tools. Since pooled samples rather than biological replicates were used, differential expression analysis between the two lines was performed by IDEG6. Target genes were identified by the TargetFinder software [71]. Functional annotation of target genes was performed using the same approach as the genes identified from RNA-seq. We conducted one biological replicate for RNA-seq and siRNA sequencing. The GenBank accession number of RNA-seq and the miRNA profiling data is SRP101680.

### 4.4. Real-Time RT-PCR

Before reverse transcription, the total RNA was treated with RNase-free DNase I (Promega, Madison, WI, USA) for 15 min to degrade the genomic DNA. Stem-loop RT-PCR was used to examine the miRNA expression level in different tissues following the procedure reported previously [72]. Primers used for stem-loop RT were designed according to the published method [73]. U6 specific primer was added simultaneously as reference for accurate normalization in each reaction. The U6 gene was selected as previously described [72]. Primers used in the miRNA qPCR are listed in Table S9. To verify target gene expression, reverse transcription was performed according to the instruction of the FastQuant RT Kit (Tiangen, Beijing, China). Semi-quantitative RT-PCR was performed as described previously using the primers listed in Table S10. The expression level of the actin gene in *B. napus* was used to standardize the RNA sample for each semi-quantitative RT-PCR. All qRT-PCR reactions were run in the CFX96 Real Time System (Bio-Rad, Hercules, CA, USA) using SYBR Green (Tiangen, China) according to the instructions. Briefly, 12.5 μL SYBR mixture, 1 μL universal reverse primer, and 1 μL specific primer were added for each reaction. Three technical replicates were performed for each sample.

## Supplementary Materials

Supplementary materials can be found at www.mdpi.com/1422-0067/18/5/887/s1.

## Abbreviations

BR	Brassinosteroid
TCP	TEOSINTE-BRANCHED/CYCLOIDEA/PCF
ARF	Auxin response factor
DEG	Differentially expressed gene
NAC	NAM, ATAF, and CUC
IAA	Indole-3-acetic acid
SULT	Sulfate Transporter
