# Supplementary material for: Integrative RNA- and miRNA-Profile Analysis Reveals a Likely Role of BR and Auxin Signaling in Branch Angle Regulation of B. napus
Source: Int J Mol Sci. 2017 May 8;18(5):887. doi: 10.3390/ijms18050887 (PMC5454811; doi:10.3390/ijms18050887)
Supplement: Supplementary file 1 [file ijms-18-00887-s001.zip › Table S1-S2.pdf]

Table S1. Primers used for semi-quantitative RT-PCR

Gene name	Forward sequence	Reverse sequence
BnaA01g35830D	CCTCTTGTAACCCTACCTCG	GGACAGGTTGATCAACAGCG
BnaA05g14370D	GGTGAGGCGGAGATAACTCA	TGCCTTCCTTCTCGCATTTC
BnaA06g14090D	TCAATTGAGGTTCTCAGCGG	GTGCCTTCTTCTCGCATTTC
BnaA08g17390D	TCCTCATGAAGTTACAGGAG	CGAACACTTGATTCTTCGGT
BnaC04g18710D	TACTCCTGTTACTGCAGATG	CTTCTGCGTCAGTTTTGGAC
BnaC08g18670D	CAACACATTGTCAGCTGGTT	AGAACAAGAAGGAGGAGCTT
BnaA02g05070D	TGACTACAGGGTGGAGTGTT	GGTGCCGCCAAGATTGTTAT
BnaC02g38080D	CTATGATTGATATCCTTGGG	CCAAATGTTCTCTTCTGAGT
BnaC09g02170D	CGATAGGTTATTGACATCAG	GGAAACTTCAGAGATTCCTAA
BnaC02g02210D	TCTGCCGATGCGGAAATGAA	TAGCTATCCGCAGAGTCTCA
BnaC02g28720D	AAACCGATCCATTCGCCGTT	CACTTGACGATGACGTAGAG
BnaA02g23290D	ACAAGAAGCTTTACAGTCATC	CATGACTTAGGTCTAAACTCA
BnaC06g39100D	CAACTTCATGAGCCCTTCTT	TCTCTCCTTCCCAAGCTTTA
BnaC08g36760D	GCTTAAAGCTCTGTGTTCCG	CCATGGCTTAACTTGGCTGT
BnaA01g25880D	TACGAGATTGTTCTAGCGGG	CTCAGATACTGCGTACTGAG
BnaA05g19350D	GTGAGCTTGTCCGTGCTACA	TGAGGCTGGAACCCACCATT
BnaC06g03450D	CAAGAGAAGAGGCCAAGAAG	CTCGGCATAGGCTGTTTCGA
BnaA06g16880D	CATAGTAGACTCCAACGTCA	TTCCATCGCAATCTCATCAG
BnaC02g38080D	GACTAGCCCTTTTGATTCGT	GTGTTTCCAAGAGTTTCTGC

BnaC09g02170D	CACAACTGATGTCAATAACC	GAGTTCTTAGAGAAGTTGGA
BnaC02g02210D	TCACCGAATAGATGCGTCAT	TTCTCATCGTTTAGAGCAGT
BnaC06g39100D	ACTCGTGGTTGCTGAACCA	TGTGGCTCCTCCAGAGTTT
BnaA01g33420D	TATGGGTTCAGTTTCTGAAG	TTCTGTTCTTGCACTTTTCC
BnaA06g02010D	CGAAGACGAAGCTGGTTCAT	CTCATCTTCTCTTGCTTGCC
BnaA06g27950D	CACAACAGCAACATCAATGG	AAACATGCAACCTCTTCACG
BnaC03g13360D	GGTTCATCTGAATCTTCACG	GGCCTTGTAGGTATTGTACG
BnaC06g09030D	GAGCCCTCACTCACCTTATT	TGATCCCAATCATCCAGTCG
BnaC08g43820D	CTGGGATTACCAGGAACAGA	TACACCAGAGCCTAAACACC
BnaC08g43830D	TCGAGCTGAATCTGAGAGAC	TCAAGCTCTGCTCTTGCACT
BnaC08g50150D	TTGAGCTGGGGAAGACTAAT	TCCAATCTCCTTCTTTATCC
BnaCnng41350D	TCGCCAGTGACTGGAACCA	CTTCAAGCTCGTACTTTTGC

Table S2. Primers used for stem loop qRT-PCR in miRNA expression analysis

Primer name	Primer sequence
319RTR1	GTCGTATCCAGTGCAGGGTCCGAGGTATTCGCACTGGATACGACAGGGAG
165RTR1	GTCGTATCCAGTGCAGGGTCCGAGGTATTCGCACTGGATACGACAGGGGG
165RTR2	GTCGTATCCAGTGCAGGGTCCGAGGTATTCGCACTGGATACGACAAGGGG
156RTR1	GTCGTATCCAGTGCAGGGTCCGAGGTATTCGCACTGGATACGACGTGCTC
160RTR1	GTCGTATCCAGTGCAGGGTCCGAGGTATTCGCACTGGATACGACTGGCAT

390RTR1	GTCGTATCCAGTGCAGGGTCCGAGGTATTCGCACTGGATACGACGGCGCT
172RTR1	GTCGTATCCAGTGCAGGGTCCGAGGTATTCGCACTGGATACGACTGCAGC
156QF1	AGCAGCCATGACAGAAGAGAGT
165QF1	AGCAGCCATCGGACCAGGCTTC
319QF1	AGTAGTCATTGGACTGAAGGGA
160QF1	AGCAGCCATGCCTGGCTCCCTG
390QF1	GCGGCGGAAAGCTCAGGAGGGA
172QF1	GCGGCGGGAGAATCTTGATGATG
Universal primer	GTGCAGGGTCCGAGGTATTC
U6s	TTGGAACGATACAGAGAAGATTAGCA
U6a	TTGGACCATTTCTCGATTTGTG
